# Transcriptomic Analysis of the Chicken MDA5 Response Genes

**DOI:** 10.3390/genes11030308

**Published:** 2020-03-13

**Authors:** Shiman Yu, Haiying Mao, Meilin Jin, Xian Lin

**Affiliations:** 1 State Key Laboratory of Agricultural Microbiology, Huazhong Agricultural University, Wuhan 430070, China; yushiman3111@webmail.hzau.edu.cn (S.Y.); maohaiying@webmail.hzau.edu.cn (H.M.); jinmeilin@mail.hzau.edu.cn (M.J.); 2 Department of Preventive Veterinary Medicine, College of Animal Medicine, Huazhong Agricultural University, Wuhan 430070, China; 3 Key Laboratory of Development of Veterinary Diagnostic Products, Ministry of Agriculture, College of Veterinary Medicine, Huazhong Agricultural University, Wuhan 430070, China; 4 The Cooperative Innovation Center for Sustainable Pig Production, Wuhan 430070, China; 5 Department of Biotechnology, College of Life Science and Technology, Huazhong Agricultural University, Wuhan 430070, China

**Keywords:** chicken MDA5, interferon stimulated genes, antiviral response, RNA-seq

## Abstract

RIG-I and MDA5 are two key pattern recognition receptors that sense RNA virus invasion, but RIG-I is absent in chickens. Although chickens have intact MDA5, the genes downstream of chicken MDA5 (chMDA5) that may mediate antiviral response are not well studied. We compared the transcriptional profile of chicken embryonic fibroblasts (DF1) transfected with chMDA5, and poly(I:C), using RNA-seq. Transfected chMDA5 and poly(I:C) in DF1 cells were associated with the marked induction of many antiviral innate immune genes compared with control. Interestingly, nine interferon-stimulated genes (ISGs) were listed in the top 15 upregulated genes by chMDA5 and poly(I:C) transfection. We used real-time PCR to confirm the upregulation of the nine ISGs, namely, MX1, IFI6, IFIT5, RSAD2, OASL, CMPK2, HELZ2, EPSTI1, and OLFML1, by chMDA5 and poly(I:C) transfection in DF1 cells. However, avian influenza virus H5N6 infection only increased MX1, IFI6, IFIT5, RSAD2, and OASL expression levels. Further study showed that the overexpression of these five genes could significantly inhibit H5N6 virus replication. These results provide some insights into the gene expression pattern induced by chMDA5, which would be beneficial for understanding and identifying innate immune genes of chicken that may lead to new antiviral therapies.

## 1. Introduction

The recognition of viruses by host cells is mediated by pattern recognition receptors (PRR) sensing virus-specific pathogen-associated nucleic acids. PRR activation leads to the induction of type I interferon (IFN), cytokine secretion, and the activation of antigen presenting cells promoting adaptive immune responses [1,2]. PRRs consist of four main categories: the retinoic acid-inducible gene I (RIG-I)-like receptors (RLR), the Toll-like receptors, the NOD-like receptor, and C-type lectin receptors. RLRs are ubiquitously expressed in the cytoplasm and comprise RIG-I, MDA5, and LGP2. RIG-I and MDA5 contain two N-terminal caspase activation and recruitment (CARD) domains [3] and a C-terminal regulatory or repressor domain. The C-terminal domain is involved in viral RNA binding, and CARD domain is required for the interaction with the CARD domain of the mitochondrial adaptor molecular MAVS [4,5]. The interaction of CARDs then activates the downstream signaling cascade, which results in the expression of type I IFNs. This condition then induces IFN-stimulated gene (ISG) expression to elucidate antiviral effect. In contrast to RIG-I and MDA5, LGP2 completely lacks the N-terminal CARDs [6], indicating that it is not able to interact with MAVS and to induce downstream signaling through CARD-CARD binding. However, LGP2 seems to serve as a regulator of the RIG-I and MDA5 signaling pathways [7,8,9]. RIG-I can recognize uncapped 5′-triphosphate (5′ppp) single-strand RNA and short blunt 5′-ppp dsRNA [10]; unlike RIG-I, MDA5 recognizes long dsRNA [11] and can be activated by the synthetic dsRNA analogue poly(I:C) [12]. Based on the feature of the recognized RNA, RIG-I plays an essential role in response to various negative-strand RNA virus. However, MDA5 senses essentially positive-strand RNA virus, particularly the *Picornaviviridae* and *Flaviviridae* members [13,14,15].

Although RLRs are generally conserved among vertebrates, the RIG-I gene cannot be identified in chicken cells [16,17]. The loss of RIG-I in chicken may account for the attenuation of the antiviral innate immune response compared with duck, which does possess the RIG-I gene. However, chicken cells have an intact MDA5 gene, which can be triggered by avian influenza virus (AIV) and Newcastle disease virus (NDV) infection [12,18,19,20]. The N-terminal 1-483 amino acids of the chMDA5 containing the CARD domain can be exploited as an efficacious adjuvant for vaccine against lethal H5N1 influenza virus and NDV [21]. Moreover, similarly to mammalian MDA5, chicken MDA5 (chMDA5) can mediate type I IFN responses in chicken cells stimulated with synthetic dsRNA. Importantly, Linger et al. demonstrated that chicken MAVS is essential to mediate chMDA5-dependent type I IFN induction downstream of chMDA5 and upstream of chIRF3 [12]. These deductions indicate that the pathway downstream of chMDA5 is intact in chickens, and chMDA5 plays a pivotal role in sensing RNA virus invasion and the induction of type I IFN. However, the mechanism of chMDA5 in the induction of type I IFN and the feature of the gene expression induced by chMDA5 remains largely unknown.

In this study, we aim to exploit the genomic resources available for chickens to identify chicken gene expression altered by chMDA5 and poly(I:C) transfection in chicken cell line DF1. We compared the transcriptional profiles in DF1 cells transfected with chMDA5, and poly(I:C) using RNA-seq. The results indicated that chMDA5 and poly(I:C) transfection significantly increased the expression of several innate antiviral genes. Some of the differentially expressed genes (DEGs) were verified by quantitative real-time PCR (qRT-PCR). We also demonstrated that several ISGs that were mostly regulated by chMDA5 could significantly inhibit AIV H5N6 infection. This study provides a foundation for further research on understanding and identifying innate immune genes of chicken that may lead to new antiviral therapies.

## 2. Materials and Methods

### 2.1. Cells and Virus

DF1 and MDCK cells were propagated in Dulbecco’s minimal essential medium (DMEM) (Hyclone) supplemented with 10% heat-inactivated fetal bovine serum (FBS) (Hyclone). DF1 cells were incubated at 39 °C, while MDCK was incubated at 37 °C in a humidified incubator with 5% CO_2_. The AIV A/duck/Hubei/WH18/2015 (H5N6) used in this study was conserved in our laboratory and propagated in the allantoic cavities of 9- to 11-day-old fertile SPF chicken eggs. Experiments with the H5N6 virus were conducted in an Animal Biosafety Level 3 laboratory, Huazhong Agricultural University, and complied with the institutional biosafety manual.

### 2.2. Construction of Expression Vectors

Total mRNA from DF1 cells was extracted by TRIzol, and 4 μg RNA was reverse transcribed using 2 Unit AMV (Takara) in 40 μL volume under the following program: 42 °C for 1 h and 72 °C for 15 min. Genes were cloned from the chicken cell cDNA into a pCMV-3Flag vector. The primers for cloning are listed in Supplementary Table S1.

### 2.3. Dual-Luciferase Reporter Assays

DF1 cells in 12-well plates were transfected with 0.5 μg empty vector, chMDA5, or 500 ng poly(I:C) together with 0.5 μg chicken IFN-β-luc and 10 ng internal control Renilla (PGL4.75 hRluc/CMV) (Promega). After transfection for 24 h, cells were lysed, and firefly and Renilla luciferase activities were measured in accordance with the instructions of the manufacturer. All obtained luciferase values were normalized against those of the Renilla luciferase control. For each assay, at least three independent experiments were performed, and each experiment was performed in triplicate.

### 2.4. RNA Sequencing

The experiments were divided into three groups: control, chMDA5, and poly(I:C). DF1 cells in each group that were seeded in a 6-well plate were transfected with 2 μg/well empty vector, chMDA5-Flag, and poly(I:C) for three replicates each using Lip8000 (Beyotime, Shanghai, China). After 24 h, total RNA was extracted using TRIzol (Invitrogen, Carlsbad, CA, USA) following the instructions of the manufacturer. The purity and concentration of the RNA from each sample were measured by a NanoDrop 2000 (Thermo Scientific, Waltham, MA, USA). A NanoPhotometer spectrophotometer was used to detect RNA purity. RNA integrity was assessed on an Agilent 2100 Bioanalyzer (Agilent Technologies, Palo Alto, CA, USA). Sequencing libraries were generated on the basis of NEBNext Ultra Directional RNA Library Prep Kit (New England Biolabs, Ipswich, MA, USA). High-quality cDNA libraries were then sequenced on an Illumina Hiseq 2500 sequencer (Illumina, San Diego, CA, USA) and the bases were called using the software CASAVA v.1.8.2 (Illumina), and generated 150 bp paired-end reads.

### 2.5. Transcriptome Analysis

High-quality data were controlled by removing poly N and low-quality reads from the raw data. After that, clean reads were acquired. The Q30 scores and GC content of the clean reads were calculated. Clean reads were then mapped to the chicken reference genome (GRCg6a) download from NCBI (https://www.ncbi.nlm.nih.gov/genome/?term=chicken) via HISAT2 (v2.0.1) software. To detect DEGs, the number of clean reads assigned to a gene was counted using HTSeq v0.6.1 [22] and then normalized to the values of fragments per kilobase of exon per million fragments mapped (FPKM) [23]. The differential expression levels among the groups were analyzed using DEseq2 R package (1.20.0) [24]. DEGs were identified by setting the corrected *p* value < 0.05 and |log2(foldchange)| > 1 as the threshold parameters. GO and KEGG of DEGs were analyzed by ClusterProfiler, with a p value less than 0.05. NetworkAnalyst (https://www.networkanalyst.ca/) was used to perform a visual analytic of protein–protein association networks based on the STRING database (v11.0), with default parameters.

### 2.6. qRT-PCR Analysis

RNA was extracted using TRIzol, followed by purification from the final aqueous phase using the RNeasy Mini Kit (Qiagen). The RNA was DNase (Promega) treated and first-strand cDNA was synthesized using All-in-one cDNA Synthesis SuperMix (Bimake). The relative mRNA expression was determined via SYBR Green-based qRT-PCR using SYBR Green supermix (Bimake) under an ABI ViiA 7 PCR system. All mRNA levels were normalized to that of *GAPDH*. Gene-specific primers for qRT-PCR are shown in Supplementary Table S2.

### 2.7. Viral Infection and Titer Determination

DF1 cells that were transfected with plasmids for 24 h were challenged with H5N6 virus at one multiplicity of infection (MOI). After 1 h of viral adsorption at 39 °C, the cells were washed twice with phosphate buffer saline (PBS) and the supernatants were replaced with DMEM medium containing 1% FBS. After that, the cells were cultured in a 39 °C humidified incubator with 5% CO_2_. At 24 h post-infection (hpi), the cells were lysed for RNA extraction and the supernatants were collected, and the viral titers were analyzed by plaque assays in MDCK cells.

### 2.8. Statistical Analysis

The results were expressed as the mean ± SD, and all data are representative of no less than three independent experiments. Data analysis was performed using Student’s *t*-test. Differences between means were considered significant at *p* values of <0.05.

## 3. Results

### 3.1. ChMDA5 and Poly(I:C) Significantly Activated Chicken IFN-β Expression

**Figure 1:**
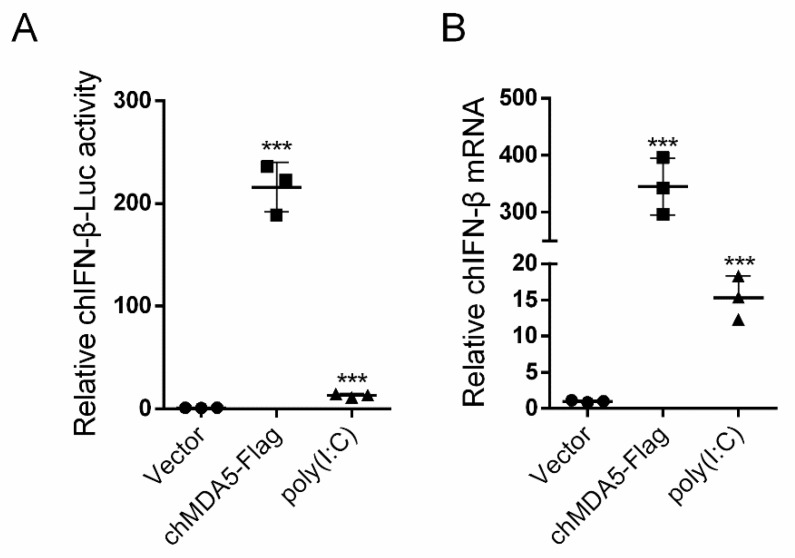
chMDA5 and poly(I:C) transfection significantly increased chIFN-β expression. (**A**) DF1 cells in 12-well plates were transfected with 0.5 μg control vector, chMDA5, or poly(I:C) together with 0.5 μg chicken IFN-β promoter reporter plasmid (chIFN-β-luc) and 10 ng control plasmid Renilla. After 24 h, cells were lysed, and firefly and Renilla luciferase activities were measured in accordance with the instructions of the manufacturer. (**B**) DF1 cells in 12-well plates were transfected with 0.5 μg control vector, chMDA5, or poly(I:C). After 24 h, total RNA was extracted, and the chIFN-β mRNA was determined by qRT-PCR. The values are shown as the mean and SD and are representative of three independent experiments. Data were analyzed using Student’s *t*-test. ***, *p* < 0.001.

We first determined if chMDA5 and poly(I:C) transfection in DF1 cells could significantly increase chicken IFN-β expression. By testing chIFN-β promotor activity using dual-luciferase report system, we fund that chMDA5 and poly(I:C) could significantly increase the chIFN-β promotor activity. ChMDA5 robustly stimulated chFN-β promotor activity more than 200-fold over control transfection; while, poly(I:C) only activated approximately 13-fold compared with control (Figure 1A). These results were validated by qRT-PCR (Figure 1B), which also indicated that chMDA5 and poly(I:C) could activate chIFN-β expression.

### 3.2. Host Response to ChMDA5 and Poly(I:C)

**Figure 2:**
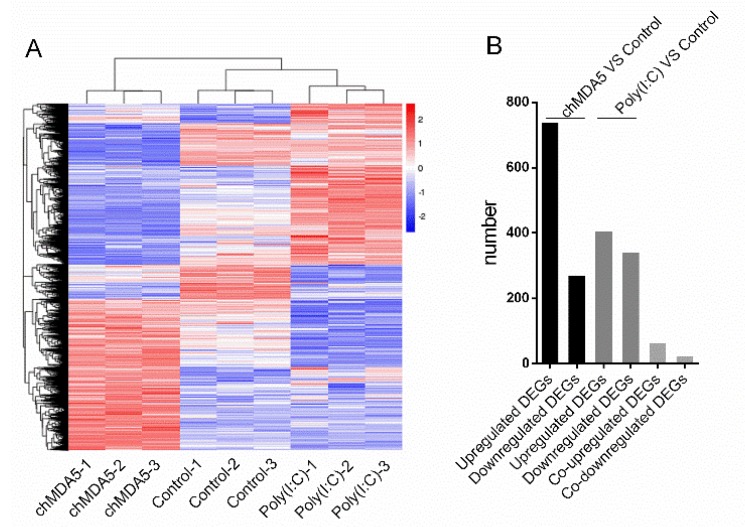
Analysis of differently expressed genes (DEGs). (**A**) Heat map analysis for classifying gene expression patterns. We took log2(FPKM + 1) for the expression of differentially expressed genes by H-cluster method and clustered them after centralized correction. The differentially expressed genes were divided into clusters, and the genes in the same cluster showed similar expression level variation trend under different treatment conditions. The *x*-axis indicates the experimental condition. Red color indicates high gene expression and blue indicates low gene expression. (**B**) Summary of the DEGs in chMDA5 and poly(I:C) group compared with control. The *x*-axis represents different terms, and the *y*-axis represents the number of DEGs.

On the basis of the results above, we extracted the RNA of DF1 cells stimulated with control vector, chMDA5, and poly(I:C). We also performed transcriptome analysis by RNA-seq. The transcripts were filtered at the cut-off *p* value < 0.05 and fold change > 1 (log2 transformed value). Under these criteria, a heat map analysis was used to classify the gene expression patterns (Figure 2A). Compared with control, 1007 genes were significantly differentially expressed in the chMDA5 transfection group. Among them, 737 genes were upregulated and 270 genes were downregulated; poly(I:C) transfection significantly altered the expression of 745 genes, of which 405 genes were significantly upregulated and 340 genes were significantly downregulated, compared with control (Figure 2B). The DEGs are provided in Supplementary Tables S3 and S4. Further analysis showed that 62 genes were upregulated in both chMDA5 and poly(I:C) groups and 23 genes were downregulated in each group (Supplementary Table S5). In the upregulated genes in both chMDA5 and poly(I:C) groups, many genes were ISGs, such as MX1, IFI6, IFIT5, RSAD2, OASL, and, CMPK2, which suggested that these genes might play important roles in restricting virus infection in chicken.

### 3.3. GO and KEGG Analysis

**Figure 3:**
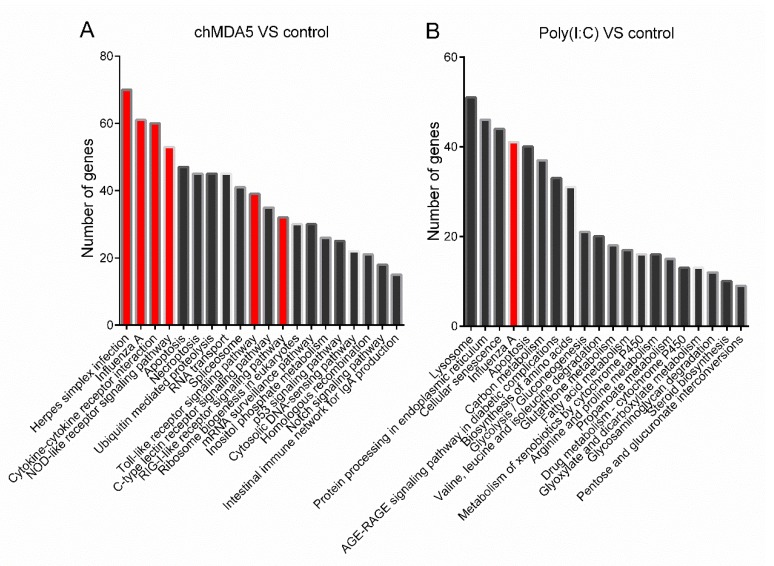
KEGG analysis of the DEGs. The top 20 KEGG pathways were significantly enriched (*p* < 0.05) in chMDA5 (**A**) and poly(I:C) (**B**) group. The red columns indicate the pathways that are associated with innate immune response. *x*-axis indicates different pathways, and *y*-axis indicates DEG numbers in the enriched KEGG pathways.

We then performed GO and KEGG analysis on the DEGs. Supplementary Tables S6 and S7 list the GO enriched biological process in chMDA5 and poly(I:C) compared with control, respectively. In the chMDA5 group, the top 20 GO terms based on the numbers of DEGs assigned to each term were mainly related to phosphorylation, biosynthetic, metabolic, and transport process (Supplementary Table S6). With regard to poly(I:C), the top 20 of GO terms were mainly related to biosynthetic and metabolic processes (Supplementary Table S7). KEGG pathway analysis was also performed to explore the function of the DEGs. Figure 3 shows the top 20 significantly enriched KEGG pathways (*p* < 0.05) based on the numbers of DEGs assigned to each term. Six of the top 20 pathways were associated with the immune-related pathway response in the chMDA5 group (Figure 3A). Among these pathways, NOD-, Toll-, and RIG-I-like receptor signaling pathways were associated with the innate antiviral immune response. In the poly(I:C) group, 12 of the top 20 pathways were associated with biosynthesis and metabolism, and one was related to influenza virus infection (Figure 3B). These results indicated that chMDA5 transfection could induce a robust immune response, and this induction was stronger than that of poly(I:C).

### 3.4. Network Analysis of the DEGs

**Figure 4:**
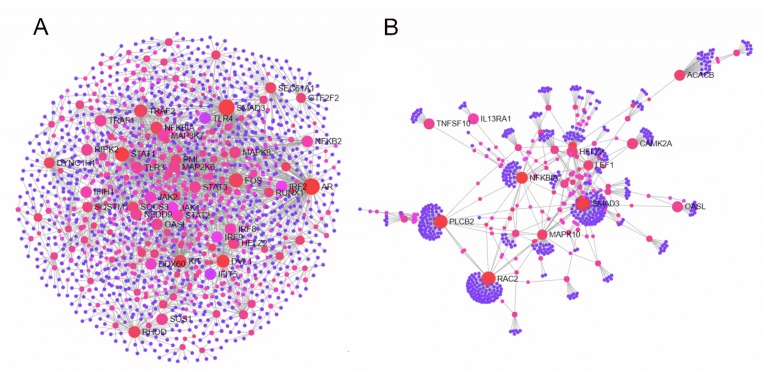
Network analysis of DEGs. NetworkAnalyst was used to construct the protein–protein network of DEGs in chMDA5 (**A**) and poly(I:C) (**B**) group based on STRING database. Nodes that connect to at least two other nodes are labeled with different red colors.

We used NetworkAnalyst to perform a visual analytic of protein–protein association networks for predicting the interaction of proteins encoded by the DEGs. In the network, nodes that connect to at least two other nodes are labeled with different red colors, and these nodes are named hubs. The more nodes that the hubs connect to, the more important the proteins may be in the corresponding process. In the chMDA5 group, many hubs were associated with immune response pathway, such as PRRs (TLR3 and TLR4), transcription factors responsible for antiviral gene expression (IRF2, IRF8, IRF9, STAT1, STAT2, NFKB1A, and NFKB), and key regulators in signaling transduction (TRAF1, TRAF2, JAK2, and RIPK2) (Figure 4A). Some hubs represented ISGs, such as OALS, IFIT5, and HELZ2, which were significantly upregulated by chMDA5 transfection. The network indicated that these hubs of proteins might play important roles in the antiviral immune response. The network constructed on the basis of DEGs after poly(I:C) treatment was less complicated (Figure 4B) than that in chMDA5. Several hubs were associated with immune and inflammatory response, such as NFKB1A, TNFSF10, IL13RA1, and MAPK10 (Figure 4B). Interestingly, OASL and HELZ2 were also in these hubs. We further analyzed the network based on the upregulated DEGs in both chMDA5 and poly(I:C) groups, which suggested that the upregulated genes were also mainly associated with immune response (Figure S1).

### 3.5. Validation of the Upregulated Genes in Both chMDA5 and poly(I:C) Groups

**Figure 5:**
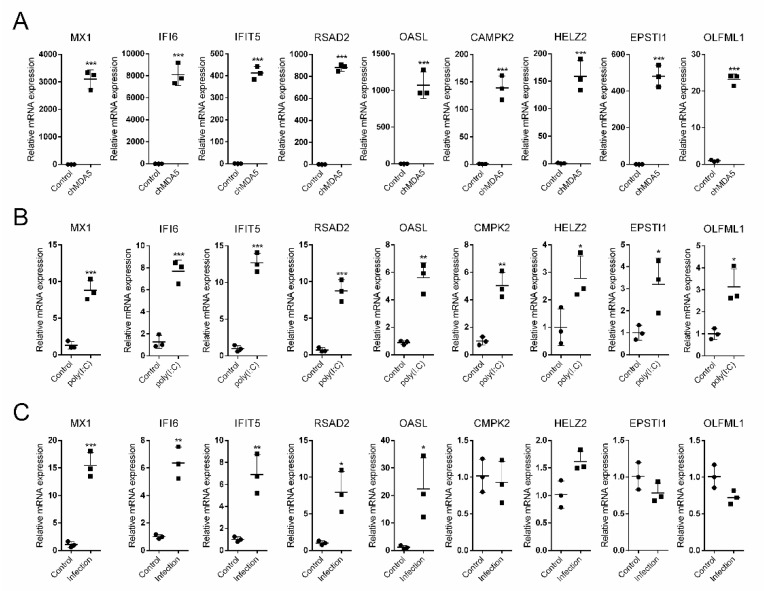
Verification of the upregulated DEGs by qRT-PCR. The total RNA of ChMDA5, poly(I:C) transfected DF1 cells (**A**,**B**), or H5N6 virus infected DF1 cells (**C**) were extracted, and the selected gene expression levels were determined by qRT-PCR. Expression of all the selected genes was normalized to GAPDH. The values are shown as the mean and SD and are representative of three independent experiments. Data were analyzed using Student’s *t* test. ***, *p* < 0.001; **, *p* < 0.01; *, *p* < 0.05.

We selected nine immune genes from the upregulated DEGs (Supplementary Table S5), namely, MX1, IFI6, IFIT5, RSAD2, OASL, CMPK2, HELZ2, EPSTI1, and OLFML1, for qRT-PCR analysis to further confirm the DEGs in the transcriptome data. These genes were also listed in the top 15 upregulated genes in both chMDA5 and poly(I:C) groups. The transcriptome data indicated that all the selected genes could be significantly upregulated by chMDA5 (Figure 5A) and poly(I:C) transfection (Figure 5B). We also investigated the effect of H5N6 virus infection on these gene expression levels (Figure 5C). H5N6 virus infection could significantly increase MX1, IFI6, IFIT5, RSAD2, and OASL expression levels but not CMPK2, HELZ2, EPSTI1, and OLFML1 expression levels.

### 3.6. Chicken MX1, IFI6, IFIT5, RSAD2, and OASL Significantly Inhibited H5N6 Virus Infection

**Figure 6:**
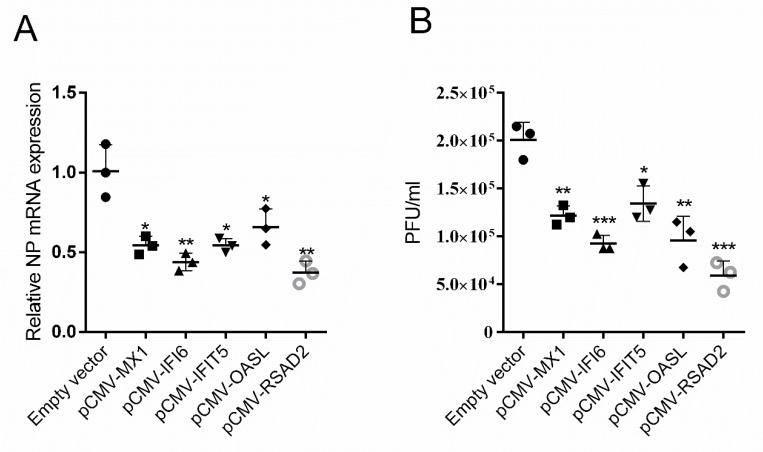
Chicken MX1, IFI6, IFIT5, RSAD2, and OASL significantly inhibited H5N6 virus infection. DF1 cells in 12-well plates were transfected with 1 μg empty vector, MX1, IFI6, IFIT5, RSAD2, and OASL expression vector. After 24 h, cells were infected with 1 MOI H5N6 virus. After 24 hpi, the cells were lysed for RNA extraction, and the supernatants were collected for the viral titers. The mRNA of viral NP was determined by qRT-PCR (**A**), and the viral titer was determined by plaque assay (**B**). The values are shown as the mean and SD and are representative of three independent experiments. Data were analyzed using Student’s t test. ***, *p* < 0.001; **, *p* < 0.01; *, *p* < 0.05.

MX1, IFI6, IFIT5, RSAD2, and OASL could be upregulated by chMDA5, poly(I:C), and H5N6 virus stimulation. Thus, we speculated that these genes could play an antiviral role. We cloned chicken MX1, IFI6, IFIT5, RSAD2, and OASL into eukaryotic expression vector and evaluated their effects on virus infection in DF1 cells. As shown in Figure 6A, MX1, IFI6, IFIT5, RSAD2, and OASL overexpression could significantly reduce nucleoprotein (NP) mRNA level of H5N6 virus in DF1 cells. Viral titer determination also demonstrated their restriction in H5N6 virus infection (Figure 6B).

## 4. Discussion

Host cells recognize invading viruses by RIG-I and MDA5 and then induce type I IFNs (IFN-α and IFN-β) to combat virus infection. IFNs’ antiviral function is mainly through the induced production of ISGs. Unlike mammals, chicken has lost RIG-I but reserves MDA5, which implies its key roles in virus recognition and downstream IFN and ISG production. However, no study has been done to reveal the pattern of chMDA5-response genes. In the present study, we aim to systematically identify chMDA5-response genes by RNA-seq through transfecting chMDA5 and dsRNA mimic poly(I:C) into chicken DF1 cells. From the transcriptome analysis, we identified several upregulated genes associated with antiviral roles, which provided a basis to further understand and identify innate immune-related genes of chicken that may lead to new antiviral therapies.

Enriched pathway analysis indicated that chMDA5 could activate several pathways associated with innate immune response, including influenza virus A, Toll-like, and RIG-I-like receptor signaling pathways, which demonstrated the intactness of the chMDA5 downstream pathway. We found that, after chMDA5 transfection, TLR3 and MDA5 were significantly upregulated. This condition would translocate downstream IFN regulatory factors to the nucleus upon phosphorylation, which would activate IFN-β production. Four IRFs (IRF1, IRF7, IRF8, and IRF9) were upregulated in chMDA5 transfection. IRF3, which is a key transcriptional factor for IFN-β in mammals, appeared to be lost in chicken cells [25]. However, IRF7 was demonstrated to be crucial in mediating IFN-β signaling in chickens [26]. However, the roles of IRF1, IRF8, and IRF9 remain to be determined. IFN-β can bind its receptor, followed by the activation of the JAK family genes, which subsequently phosphorylates STAT1 and STAT2 [27,28], which then translocate into the nucleus in the form of heterodimer and induce the transcription of ISGs. In this study, we found that STAT1 and STAT2 were significantly increased by chMDA5 transfection, which could lead to the increased expression of ISGs. As expected, we identified several ISGs after chMDA5 and poly(I:C) transfection. We focused on the upregulated DEGs in both chMDA5 and poly(I:C) groups, and found that within these MX1, IFI6, IFIT5, RSAD2, OASL, CMPK2, HELZ2, EPSTI1, and OLFML1 were ISGs (Table S5), which were all validated by qRT-PCR as being increased upon chMDA5 transfection by qRT-PCR. Although the inducing capacity of IFN-β in DF1 cells by poly(I:C) was less than that in chMDA5 transfection, these ISGs were also upregulated to some different extents in poly(I:C)-stimulated DF1 cells (Figure 5). Interestingly, H5N6 virus infection also increased RSAD2, OASL, IFIT5, MX1, and IFI6 expression levels but not CMPK2, HELZ2, EPSTI1, and OLFML1 expression levels. This finding suggested that H5N6 virus infection could stimulate various types of ISG expression, or H5N6 virus could inhibit some ISG expression through its proteins, such as NS1.

The antiviral effect of IFN-β is mainly dependent on ISG expression. Theoretically, the ISGs upregulated by chMDA5 and poly(I:C) could restrict virus infection. Interestingly, RSAD2, OASL, and MX1 were also reported to be significantly increased by NDV infection in DF1 cells [19]; IFIT5, OASL, and MX1 could also be increased in duck RIG-I transected DF1 cells that were infected by AIV [18]. RSAD2 is an evolutionary conserved protein that can restrict various viruses, including measles virus [29], Enterovirus [30], and influenza virus [31]. MX1 protein confers antiviral function in transfected cells and transgenic animals [32] and can protect against lethal infection by influenza virus in a mouse model [33]. Human OASL lacks oligoadenylate synthetase (OAS) activity but can inhibit RNA virus proliferation. Chicken OASL encodes a typical domain of OAS but has two ubiquitin-like domains, which are necessary for its antiviral role [34,35]. IFIT5, which is a member of the IFN-induced proteins with tetratricopeptide repeats family, can sequester viral RNA transcript in a multi-protein complex in mammalian cells [36]. Whether these proteins could play an antiviral function in DF1 cells should be investigated. We cloned chicken MX1, IFI6, IFIT5, RSAD5, and OASL and found that these genes could significantly inhibit H5N6 virus infection in DF1 cells. However, the mechanisms should be further determined.

We did not investigate the roles of chicken CMPK2, HELZ2, EPSTI1, and OLFML1 in influenza virus infection. However, they might still have antiviral effects in chickens, because of their antiviral effects in other species [37,38,39,40,41]. Notably, HELZ2, which is a helicase with a zinc finger, is in the protein hub that is connected to many hits in the networks based on the upregulated DEGs in both chMDA5 and poly(I:C) groups. It was reported that HELZ2 was an interferon effector mediating suppression of Dengue virus. This implies that HELZ2 may have a potential role in the antiviral response in chickens [41]. The specific roles of these five investigated chicken genes on virus infection require further investigation.

## 5. Conclusions

We used RNA-seq to investigate the features of the chMDA5-response genes by transfection of chMDA5 and poly(I:C). We identified many DEGs that were associated with immune response. Interestingly, some DEGs that were listed as top 10 upregulated genes were ISGs in chMDA5 transfection, namely, RSAD2, MX1, IFIT5, IFI6, and OASL. These genes exhibited significant antiviral effect against AIV. Thus, our study provides a pool of chMDA5-response genes available for antiviral strategy development in the future.

## Supplementary Materials

The following are available online at https://www.mdpi.com/2073-4425/11/3/308/s1, Figure S1: The network analysis of upregulated genes in both chMDA5 and poly(I:C) groups, Table S1: The primers used for cloning, Table S2: The primers used for qRT-PCR, Table S3: Differentially expressed genes in chMDA5 compared with control, Table S4: Differentially expressed genes in poly(I:C) compared with control, Table S5: Genes similarly regulated in both chMDA5-N and Poly(I:C) groups compared with control, Table S6: The enriched GO biological process in chMDA5 compared with control, Table S7: The enriched GO biological process in poly(I:C) compared with control.
