# Supplementary material for: Transcriptomic Analysis of the Chicken MDA5 Response Genes
Source: Genes (Basel). 2020 Mar 13;11(3):308. doi: 10.3390/genes11030308 (PMC7140832; doi:10.3390/genes11030308)
Supplement: Supplementary file 1 [file genes-11-00308-s001.zip › Supplementary materials/Supplementary Table S1.docx]

| Primers | Sequence (5'→3') | Enzyme |
| --- | --- | --- |
| MDA5-F | ATATCGATATGTCGGAGGAGTGCCGAGACGA | ClaI |
| MDA5-R | ATGCTAGCTTAATCTTCATCACTTGAAGGACAATGAGATG | NheI |
| IFI6-F | CCAAGCTTATGTCTGACCAGAACGTCCACAAAGC | HindIII |
| IFI6-R | GCTCTAGAGCGCCTTCCTCCTTTGCCACC | XbaI |
| MX1-F | CCATCGATATGAACAATCCATGGTCCAACTTCAGCT | ClaI |
| MX1-R | GCTCTAGACAGAGACTTAAAGTCTACCAGGTATTGGTAGGC | XbaI |
| OASL-F | CCAAGCTTATGGGGTTGGAGAGCGTGAGCTC | HindIII |
| OASL-R | GCTCTAGAGGAGGGCACGCAGCGTCTG | XbaI |
| IFIT5-F | AAATCGATATGAGTACCATTTCCAAGAATTCCTTGAAGA | ClaI |
| IFIT5-R | AAGGTACCGAGCTTGAGAGGGAAAGTCGTAGCTCCA | KpnI |
| RSAD2-F | ATGAATTCATGCTGCTGGGCGTTCTGGATCA | EcoRI |
| RSAD2-R | AATCTAGACCAGTCCAGAATCATGTCTGCTTTACTCCA | XbaI |

Table S1. Primers for cloning

Note: the base-pare underline indicates restriction enzyme cutting site.
