# Supplementary material for: Transcriptomic Analysis of the Chicken MDA5 Response Genes
Source: Genes (Basel). 2020 Mar 13;11(3):308. doi: 10.3390/genes11030308 (PMC7140832; doi:10.3390/genes11030308)
Supplement: Supplementary file 1 [file genes-11-00308-s001.zip › Supplementary materials/Supplementary Table S2.docx]

| Primers | Sequence (5'→3') |
| --- | --- |
| IFN-β-F | AGCTCTCACCACCACCTTCTC |
| IFN-β-R | TGGCTGCTTGCTTCTTGTCCTT |
| MX1-F | AAGCCTGAGCATGAGCAGAA |
| MX1-R | TCTCAGGCTGTCAACAAGATCAA |
| OASL-F | ACATCCTCGCCATCATCGA |
| OASL-R | GCGGACTGGTGATGCTGACT |
| RSAD2-F | CCCCGGGAGGACAAGGACGAGAC |
| RSAD2-R | CCAGGGGCAGCACGAAGGAGGTC |
| IFI6-F | CAGCAGTGGGATCTCCGGTGGC |
| IFI6-R | GCGCCTTCCTCCTTTGCCACCCA |
| IFIT5-F | TGCTCTGAGGGAAGAACCCAACA |
| IFIT5-R | AGGCTCCAGGGATGAGTCCACTT |
| HELZ2-F | AGCCACCACGGAGAAGACAATCT |
| HELZ2-R | CTCATCCAACAGGCGGTCAACAG |
| EPSTI1-F | TGGAGGGAAGGCAGGTCATACG |
| EPSTI1-R | TGCTGCTCTTGCTGTTGATGGT |
| CMPK2-F | GGTGCTGGACATCCTGGAGAAGT |
| CMPK2-R | GCTGGCGGAGACCTTAACAGAAC |
| OLMFL1-F | TGCAAGATGCAGCACTGATGAACT |
| OLMFL1-R | GTCCTGACAGACGTGACAGTACCA |

Table S2 Primers for qRT-PCR
